# Fabrication of Crack-Free Nickel-Based Superalloy Considered Non-Weldable during Laser Powder Bed Fusion

**DOI:** 10.3390/ma11081288

**Published:** 2018-07-25

**Authors:** Oscar Sanchez-Mata, Xianglong Wang, Jose Alberto Muñiz-Lerma, Mohammad Attarian Shandiz, Raynald Gauvin, Mathieu Brochu

**Affiliations:** Department of Mining and Materials Engineering, McGill University, 3610 University Street, Wong Building, Montreal, QC H3A 0C5, Canada; oscar.sanchezmata@mail.mcgill.ca (O.S.-M.); xianglong.wang@mail.mcgill.ca (X.W.); jose.muniz@mail.mcgill.ca (J.A.M.-L.); mohammad.attarianshandiz@mail.mcgill.ca (M.A.S.); raynald.gauvin@mcgill.ca (R.G.)

**Keywords:** nickel alloys, Hastelloy X alloy, additive manufacturing, microstructure, scanning electron microscopy (SEM), laser powder bed fusion (LPBF)

## Abstract

Crack-free Hastelloy X fabricated through laser powder bed fusion (LPBF) from powder with a standard chemical composition is reported. Electron backscatter diffraction (EBSD) analysis evidenced columnar grains parallel to the building direction. The typical LPBF columnar dendrite microstructure was found to be finer than reported elsewhere. Mo-enriched carbides (~50 nm), presumed to play an important role in the cracking behavior of the alloy, were confirmed along interdendritic regions. Crack-free condition was maintained after heat treatment at 1177 °C for 1 h followed by water quenching, and the resulting microstructure was analyzed.

## 1. Introduction

Laser Powder Bed Fusion (LPBF) of Ni-based Superalloys is finding applications in many fields such as aerospace and land-based turbine engines [1]. This process involves the rastering of a focused beam to selectively melt powder in a layer-by-layer methodology [2]. Unfortunately, LPBF of many of these alloys face the important issue of cracking through a variety of mechanisms, such as liquation and solidification cracking or strain-age and ductility-dip cracking [3]. The welding field has developed criteria to evaluate the cracking susceptibility of alloys [4,5]. Such concepts are often referred in the context of additive manufacturing (AM), but recent experimental evidences have shown that the cracking susceptibility is more severe in the context of LPBF, i.e., a crack-free alloy during welding can develop crack during AM [6,7]. An interesting example is Hastelloy X, a Ni-Cr-Fe-Mo solid solution strengthened alloy [8] that although categorized as a ‘weldable’ alloy [9], has been shown to be prone to hot cracking during LPBF [10,11,12,13,14].

Various explanations and strategies to eliminate cracking have been reported. Wang, et al. [10] attributed the formation of cracks in samples built using a powder containing 0.78% Si, 0.69% Mn, and 0.009% C to the residual stresses developing during processing, but were not able to recommend an optimal process window to fabricate crack-free samples. Another approach to eliminate cracking included the control of the chemical composition of the feedstock powder. Tomus, et al. [12,13] highlighted that the microsegregation of Si and C contribute to hot cracking and suggested that a composition of 0.11% Si, <0.01% Mn, and 0.01% C would inhibit crack formation, as opposed to an alloy possessing a higher concentration of these three elements. Harrison, et al., suggested that increasing the concentration of substitutional solid solution strengthening elements like Mo, Co and W helps to increase the resistance to cracking. Their experiments were carried out using a powder containing 0.31% Si, 0.22% Mn, and 0.054% C, and results showed crack density reduction compared to the original composition of 11.6 ± 2.4 to 1.6 ± 0.9 cracks per mm^2^ [14]. Recently, Marchese proposed that the formation of intergranular Mo-enriched carbides, coupled with high thermal residual stresses, resulted in hot cracking during LPBF of Hastelloy X [15].

Based on these results, the AM community has been focusing on tailoring the chemical composition of Hastelloy X as a viable avenue to mitigate cracking. A consensus in literature points towards intergranular low melting point eutectics to be responsible for the cracking susceptibility of this alloy. This has led to the commercial availability of Hastelloy X alloys with lower contents of minor elements such as Mn, Si and C, and in some cases offering alloys with a C content below specification. This approach can have, however, detrimental effects on the product’s properties; for instance, it has been shown that C content on a Ni-Cr-Fe-Mo alloy has a significant effect on its thermal fatigue properties [16]. This present work reports the fabrication of crack-free Hastelloy X samples made from powder with a standard chemical composition, and where the sound microstructure survived high temperature heat treatment.

## 2. Materials and Methods

Commercially available gas-atomized Hastelloy X powder provided by LPW Technology Ltd. with a composition displayed in Table 1 was processed using a Renishaw AM400. The powder particles were spherical in morphology, having a particle size distribution D10 = 19 µm, D50 = 41 µm, and D90 = 62 µm. Cubic samples of 1 cm^3^ were produced under Ar atmosphere and analyzed in the as-built and annealed condition. For protection of proprietary information, specific process parameters are not included. Cross-sections of samples were prepared for microstructure evaluation using traditional metallography techniques. Electroetching was performed using a 10% oxalic acid solution and 6 V for 5–10 s. The density of the as-built sample was analyzed using a Nikon light optical microscope (OM) equipped with a Clemex Vision System. Microstructures were observed using a Hitachi SU3500 scanning electron microscope (SEM) equipped with an electron backscatter diffraction (EBSD) detector for texture and grain morphology analysis. High magnification micrographs and electron dispersive spectrometer (EDS) line scans were obtained using a Hitachi SU8000 field emission scanning electron microscope (FE-SEM), prior to which the sample’s surface was further prepared using ion beam milling. Heat treatment was carried out under an Ar atmosphere at 1175 °C for 1 h followed by water quenching.

## 3. Results and Discussion

**Figure 1:**
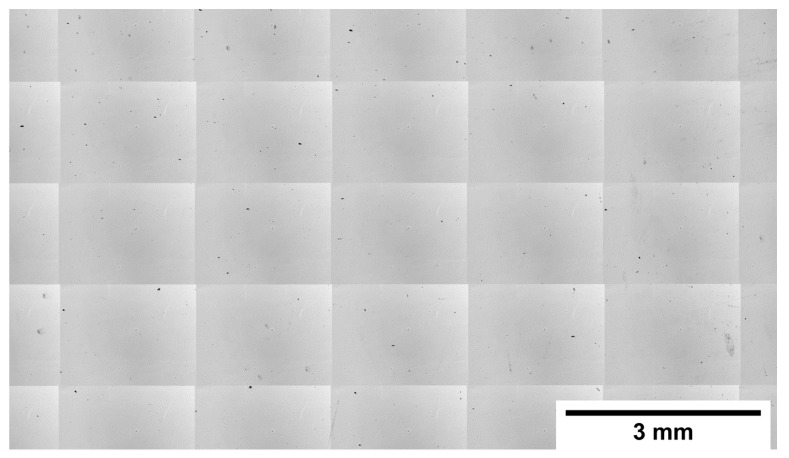
Representative optical micrograph of stitched 50× images from the cross-section of an as-built cubic sample from its polished state.

A mosaic stitched from optical micrographs of the X-Z cross-section of a cubic sample in the as-built condition taken from its polished state is shown in Figure 1. As depicted, crack-free specimens with optical density of 99.76 ± 0.11 were obtained. The remaining porosity, with irregular and spherical morphology, is homogeneously distributed within the sample and attributed to lack of fusion and gas entrapment.

**Figure 2:**
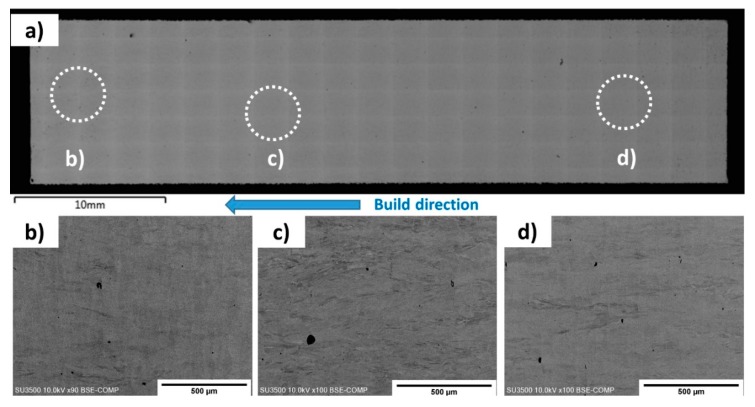
(**a**) SEM montage of the cylindrical sample and higher magnification images of the (**b**) top, (**c**) middle, and (**d**) bottom of the sample. The regions where (**b**–**d**) were taken from are highlighted on the montage.

In order to demonstrate the absence of cracks in a larger building volume, a cylindrical sample of radius 1 cm and 5 cm in height was built, vertically cross-sectioned, polished and imaged. An SEM micrograph is shown in Figure 2a, and higher magnification observation from the (b) top, (c) middle, and (d) bottom of the sample further demonstrate a crack-free cross-section.

**Figure 3:**
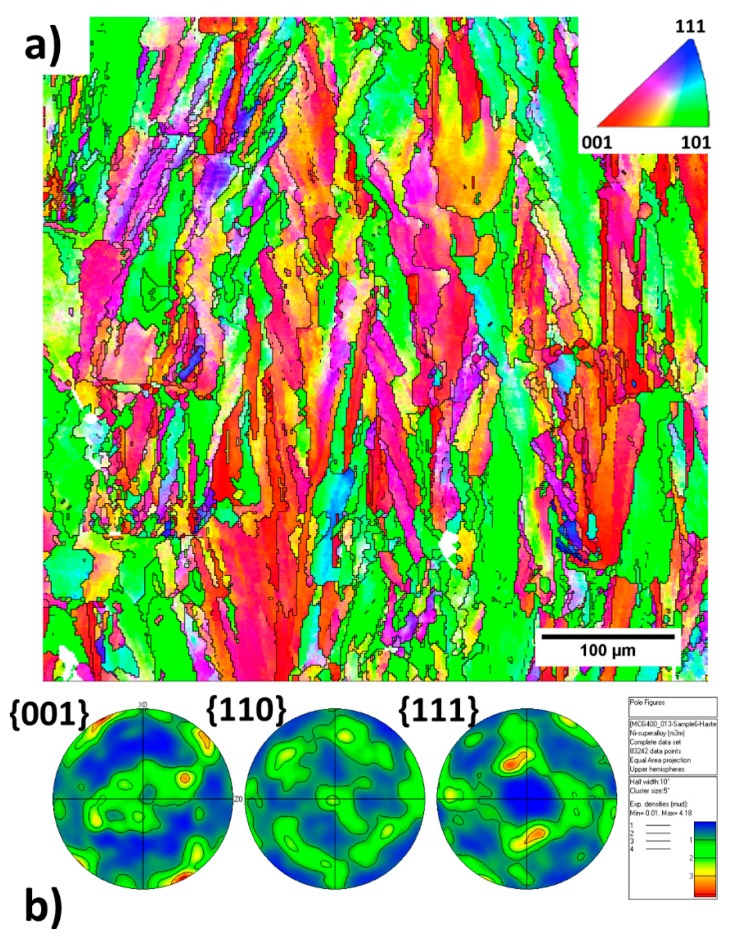
(**a**) Electron backscatter diffraction (EBSD) map taken from the central region of the as-built sample and (**b**) its corresponding pole figure.

Figure 3 shows the EBSD inverse pole figure (IPF) maps along with the corresponding pole figures collected from the cross-section of the cubic sample parallel to the build direction of the as-built condition. Columnar grains with measured widths of 9.76 ± 8.8 µm oriented towards the build direction are evident. The grains grow across several layers without changing their crystallographic orientations, indicating epitaxial growth. As shown in Figure 3a, most columnar grains show either <001> or <110> orientations. It is noted that some of the grains are not perfectly aligned with build direction. As depicted in the {001} pole figure in Figure 3b, the clusters of {001} poles near the center of the pole figure are not concentrated, indicating some angular deviations between the <001> oriented grains and build directions. On the other hand, the grains oriented in the <110> direction exhibit a weak partial fiber type feature, as observed in the {110} pole figure in Figure 3b.

**Figure 4:**
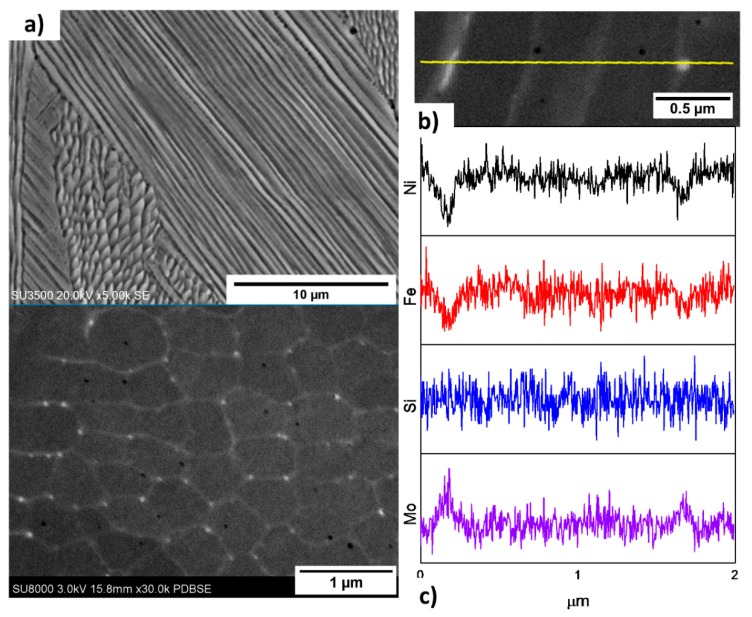
(**a**) SEM micrograph showing the typical columnar microstructure obtained from as-built LPBF Hastelloy X; (**b**) High magnification SEM micrograph; (**c**) Electron dispersive spectrometer (EDS) line scans corresponding to the region displayed above.

Figure 4a illustrates the typical fine columnar microstructure observed in the XZ plane of the sample in the as-built condition. This microstructural morphology is commonly found in the additive manufacturing processes and has been widely reported in LPBF of nickel-based superalloys, including Hastelloy X [13,14,15] as well as Inconel 625 [18,19,20,21], Inconel 718 [22,23], CM247 [7] and Inconel 738 [24,25].

The primary dendrite arm spacing (PDAS) obtained by image analysis of high magnification micrographs, corresponds to 0.4 ± 0.06 μm. An estimation of the cooling rate of the process based on the equation PDAS = aɛ^−b^, where a ≈ 50 (K/s) and b ≈ 0.33 [21], indicates that cooling conditions of 1~2 × 10^6^ K/s were achieved. When comparing with the literature data, the PDAS of the current work was found to be smaller. For example, Harrison, et al. [14] observed a similar solidification structure with PDAS ranging between 0.9 and 1.2 μm, Marchese, et al. [15] reported a PDAS of 0.65 ± 0.25 μm, while Tomus showed fine parallel dendrites with a width of 0.53 ± 0.09 μm, calculated from the reported micrographs [26].

The microstructure at higher magnification obtained through FE-SEM is shown in Figure 4b. Boundaries of the cellular structure, which corresponds simply to a different cross-section of the previously described fine columnar features, are clearly visible with nanometric precipitates dispersed along the cell boundaries. Nanometric black dots are also distinguishable in some cell cores and intercellular regions, which are attributed to gas entrapment pores.

Figure 4c displays the EDS line scan spectra crossing from left to right, a precipitate at a cell boundary, three complete cells and their boundaries, and a precipitate lying on the last boundary. Ni and Fe, which are γ matrix elements, are distinctly depleted on the precipitate. On the other hand, the presence of Mo on the same regions causes a distinguishable high-count region on the spectrum, evidencing the fact that these are Mo-enriched precipitates. Furthermore, Si, which is another element that has been suggested to segregate, appears to have no clear concentration gradient throughout the scan.

The size of the Mo enriched precipitates was measured to be 55.4 ± 4.4 nm. Marchese, et al. [15] report the formation of globular and elongated M_6_C or M_n_C_m_ Mo-enriched carbides with sizes around 100–500 nm located on interdendritic regions, along with smaller (<100 nm) unidentified precipitates within the dendrites. The authors indicated that these intercellular carbides are present within cracks and thus play a significant role in this alloy’s cracking mechanism. In welding of Hastelloy X, previous studies have shown that M_6_C carbides are abundant and enriched with Mo, and that they form a liquid film of this lower melting eutectic which is linked to hot cracking of the alloy [27].

**Figure 5:**
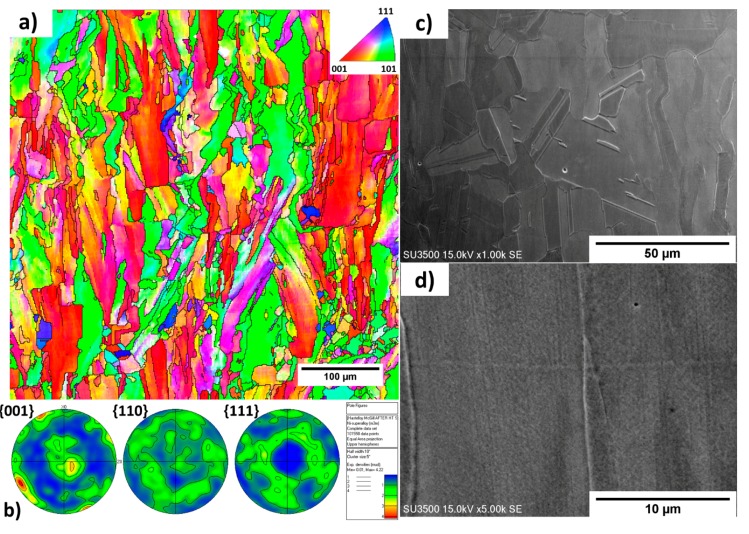
(**a**) EBSD map taken from the central region of the sample after heat treatment; and (**b**) its corresponding pole figure; SEM micrographs taken at (**c**) 5000× and (**d**) 1000× showing the microstructure of the sample after heat treatment.

Heat treatment was performed on the samples to evaluate any possible change in microstructure. Annealing of Hastelloy X has been done at 1177 °C for an equivalent of 1 h per inch of thickness [28]. After heat treatment, the resulting microstructure is shown in Figure 5. From Figure 5a, the morphology and size of the grains is not significantly altered compared with the as-built sample. Columnar grains with a measured width of 9.54 ± 8.5 µm have been observed. As for the texture, it has been observed from the {001} pole figure (Figure 5b) that the {001} poles at the center become less scattered compared with those in the as-built condition. The texture maxima close to the center of the pole figure indicates a stronger <001> texture towards build direction after heat treatment. Meanwhile, the <110> partial fibre type texture along build direction is further weakened and becomes more diffused compared with that in the as-built condition, suggesting the decreasing <110> grains along build direction after heat treatment. No clear signs of alterations to the as-built columnar grain structure are visible from the EBSD analysis; furthermore, the microstructure depicted in Figure 5c, shows signs of twinning within the sample. Twin formation has been observed in annealing of other additively manufactured superalloys, and attributed to residual stresses from the as-built condition being partially released by forming twin grains during this heat treatment [29]. At higher magnification (Figure 5d), it is clear that the columnar structure visible in the as-built microstructure are almost homogenized. Although no conclusions about segregation in the interdendritic regions can be made from the line scans in Figure 4c, the FE-SEM micrograph in Figure 3b is a backscattered electrons (BSE) image, in which contrast is obtained by differences in composition; thus, it is possible that segregating elements diffuse into the matrix during heat treatment, causing the dendritic structure to disappear.

Etter et al. [30], reported significant grain coarsening and the presence of equiaxed grains after heat treatment at 1200 °C for 4 h, and the complete disappearance of the columnar grain structure from the as-built microstructure. Marchese [31], from a fine LPBF columnar microstructure, obtained a microstructure composed of equiaxed grains and twins after a solution treatment at 1175 °C for up to an hour. He also reported that a heat treatment at 1066 °C for 1 h was ineffective in modifying the microstructure. Finally, a heat treatment performed by Tomus [26] 1175 °C for 2 h followed by air cooling managed to dissolve the as-built dendrites, but columnar grains elongated along the building direction were still observed and attributed to the absence of recrystallization occurring after heat treatment. The heat treatment schedule used here corresponds to the solutionizing treatment time and temperature used for Hastelloy X [28], so any microsegregation present would be expected to homogenize through the microstructure. Although there is a lack of agreement in literature on whether this heat treatment will fully recrystalize and eliminate the as-built columnar grains, and data obtained from EBSD (Figure 4a) does not clearly demonstrate the presence of recrystalized grains, the appearance of annealing twins visible on the studied sample is an indication that recrystallization may have occurred at least partially, since twins have been shown to form mostly during the recrystallization process [32], and have been observed in heat treated samples of IN718 processed through laser metal deposition with different degrees of recrystallization [33].

## 4. Conclusions

This work has reported the fabrication of crack-free Hastelloy X samples made from powder with a standard chemical composition through LPBF, observable on optical micrographs. Columnar grains parallel to the building directions were evidenced through EBSD in the as-built condition. Likewise, a typical microstructure for LPBF Ni-based superalloys was found, with smaller columnar structure size than reported elsewhere in literature. The presence of Mo-enriched carbides (~50 nm) was confirmed through EDS line scans obtained using an FE-SEM; these Mo-enriched carbides are presumed to play an important role in the cracking mechanism of this alloy. A subsequent heat treatment at 1177 °C for 1 h followed by water quenching was performed, after which the crack-free microstructure was sustained. After EBSD, a columnar grain structure was demonstrated to be present, and SEM micrographs displayed the presence of annealing twins and a disappearance of the as-built columnar microstructure.

## Figures and Tables

**Table 1:** Comparison of the composition of Hastelloy X from literature.

Reference	Ni	Cr	Fe	Mo	Co	W	Mn	Si	C
Wang, et al. [10]	Bal.	20.6	18.4	8.8	1.3	0.62	0.69	0.78	0.009
Harrison, et al. [14]	Bal.	21.8	18.6	9.4	1.8	1.05	0.22	0.31	0.054
Tomus, et al. [13]	Bal.	21.4	18.4	8.8	1.8	0.86	<0.01	0.11	0.01
Marchese, et al. [15]	Bal.	21.7	18.6	9.2	1.8	0.90	-	0.36	0.056
This work	Bal.	21.2	17.6	8.8	2.0	NM	<0.1	0.20	0.06
ASTM B435 [17]	Bal.	20.5–23	17–20	8–10	0.5–2.5	0.2–1	<1	<1	0.05–0.15

## References

[1] DebRoy T., Wei H.L., Zuback J.S., Mukherjee T., Elmer J.W., Milewski J.O., Beese A.M., Wilson-Heid A., De A., Zhang W. (2018). Additive manufacturing of metallic components—Process, structure and properties. Prog. Mater. Sci..

[2] Frazier W.E. (2014). Metal Additive Manufacturing: A Review. J. Mater. Eng. Perform..

[3] Chauvet E., Kontis P., Jägle E.A., Gault B., Raabe D., Tassin C., Blandin J.-J., Dendievel R., Vayre B., Abed S. (2018). Hot cracking mechanism affecting a non-weldable Ni-based superalloy produced by selective electron Beam Melting. Acta Mater..

[4] DuPont J.N., Lippold J.C., Kiser S.D. (2009). Welding Metallurgy and Weldability of Nickel-Base Alloys.

[5] Kou S. (2003). Solidification and Liquation cracking issues in welding. JOM.

[6] Attallah M.M., Jennings R., Wang X., Carter L.N. (2016). Additive manufacturing of Ni-based superalloys: The outstanding issues. MRS Bull..

[7] Carter L.N., Attallah M.M., Reed R.C. (2012). Laser Powder Bed Fabrication of Nickel-Base Superalloys: Influence of Parameters; Characterisation, Quantification and Mitigation of Cracking. Superalloys.

[8] Zhao J.-C., Larsen M., Ravikumar V. (2000). Phase precipitation and time–temperature-transformation diagram of Hastelloy X. Mater. Sci. Eng. A.

[9] Catchpole-Smith S., Aboulkhair N., Parry L., Tuck C., Ashcroft I.A., Clare A. (2017). Fractal scan strategies for selective laser melting of ‘unweldable’ nickel superalloys. Addit. Manuf..

[10] Wang F., Wu X.H., Clark D. (2011). On direct laser deposited Hastelloy X: Dimension, surface finish, microstructure and mechanical properties. Mater. Sci. Technol..

[11] Wang F. (2011). Mechanical property study on rapid additive layer manufacture Hastelloy® X alloy by selective laser melting technology. Int. J. Adv. Manuf. Technol..

[12] Tomus D., Jarvis T., Wu X., Mei J., Rometsch P., Herny E., Rideau J.F., Vaillant S. (2013). Controlling the Microstructure of Hastelloy-X Components Manufactured by Selective Laser Melting. Phys. Procedia.

[13] Tomus D., Rometsch P.A., Heilmaier M., Wu X. (2017). Effect of minor alloying elements on crack-formation characteristics of Hastelloy-X manufactured by selective laser melting. Addit. Manuf..

[14] Harrison N.J., Todd I., Mumtaz K. (2015). Reduction of micro-cracking in nickel superalloys processed by Selective Laser Melting: A fundamental alloy design approach. Acta Mater..

[15] Marchese G., Basile G., Bassini E., Aversa A., Lombardi M., Ugues D., Fino P., Biamino S. (2018). Study of the Microstructure and Cracking Mechanisms of Hastelloy X Produced by Laser Powder Bed Fusion. Materials.

[16] An N., An Y., Fan Q., Fu Z.M., Li Z.R., Zhang Y.Y. (2016). Effect of Carbon on the Microstructural Evolution and Thermal Fatigue Behavior of a Ni-Base Superalloy. Mater. Sci. Forum.

[17] Standard Specification for UNS N06002, UNS N06230, UNS N12160, and UNS R30556 Plate, Sheet, and Strip. http://www.htpipe.com/d/files/plate-material-grade/astm-b435.pdf.

[18] Marchese G., Garmendia Colera X., Calignano F., Lorusso M., Biamino S., Minetola P., Manfredi D. (2017). Characterization and Comparison of Inconel 625 Processed by Selective Laser Melting and Laser Metal Deposition. Adv. Eng. Mater..

[19] Lass E.A., Stoudt M.R., Williams M.E., Katz M.B., Levine L.E., Phan T.Q., Gnaeupel-Herold T.H., Ng D.S. (2017). Formation of the Ni3Nb δ-Phase in Stress-Relieved Inconel 625 Produced via Laser Powder-Bed Fusion Additive Manufacturing. Metall. Mater. Trans. A.

[20] Li C., White R., Fang X.Y., Weaver M., Guo Y.B. (2017). Microstructure evolution characteristics of Inconel 625 alloy from selective laser melting to heat treatment. Mater. Sci. Eng. A.

[21] Li S., Wei Q., Shi Y., Zhu Z., Zhang D. (2015). Microstructure Characteristics of Inconel 625 Superalloy Manufactured by Selective Laser Melting. J. Mater. Sci. Technol..

[22] Jia Q., Gu D. (2014). Selective laser melting additive manufacturing of Inconel 718 superalloy parts: Densification, microstructure and properties. J. Alloys Compd..

[23] Tian Y., Muñiz-Lerma J.A., Brochu M. (2017). Nickel-based superalloy microstructure obtained by pulsed laser powder bed fusion. Mater. Charact..

[24] Cloots M., Kunze K., Uggowitzer P.J., Wegener K. (2016). Microstructural characteristics of the nickel-based alloy IN738LC and the cobalt-based alloy Mar-M509 produced by selective laser melting. Mater. Sci. Eng. A.

[25] Kunze K., Etter T., Grässlin J., Shklover V. (2015). Texture, anisotropy in microstructure and mechanical properties of IN738LC alloy processed by selective laser melting (SLM). Mater. Sci. Eng. A.

[26] Tomus D., Tian Y., Rometsch P.A., Heilmaier M., Wu X. (2016). Influence of post heat treatments on anisotropy of mechanical behaviour and microstructure of Hastelloy-X parts produced by selective laser melting. Mater. Sci. Eng. A.

[27] Savage W.F., Krantz B.M. (1971). Microsegregation in Autogenous Hastelloy X Welds. Weld. Res. Suppl..

[28] Donachie M.J., Donachie S.J. (2002). Superalloys: A Technical Guide.

[29] Dinda G.P., Dasgupta A.K., Mazumder J. (2009). Laser aided direct metal deposition of Inconel 625 superalloy: Microstructural evolution and thermal stability. Mater. Sci. Eng. A.

[30] Etter T., Kunze K., Geiger F., Meidani H. (2015). Reduction in mechanical anisotropy through high temperature heat treatment of Hastelloy X processed by Selective Laser Melting (SLM). IOP Conf. Ser. Mater. Sci. Eng..

[31] Marchese G., Biamino S., Pavese M., Ugues D., Lombardi M., Vallillo G., Fino P. Heat Treatment Optimization of Hastelloy X Superalloy Produced by DMLS. https://iris.polito.it/handle/11583/2646135.

[32] Rollett A.D., Rohrer G.S., Humphreys F.J. (2017). Recrystallization and Related Annealing Phenomena.

[33] Sui S., Zhong C., Chen J., Gasser A., Huang W., Schleifenbaum J.H. (2018). Influence of solution heat treatment on microstructure and tensile properties of Inconel 718 formed by high-deposition-rate laser metal deposition. J. Alloys Compd..

